# A Two-Step Filtering Approach for Indoor LiDAR Point Clouds: Efficient Removal of Jump Points and Misdetected Points

**DOI:** 10.3390/s25195937

**Published:** 2025-09-23

**Authors:** Yibo Cao, Yonghao Huang, Junheng Ni

**Affiliations:** School of Artificial intelligence, South China Normal University, Foshan 528000, China; reancolstark@163.com (Y.H.);

**Keywords:** simultaneous localization and mapping, indoor mobile robot, point cloud filtering algorithm, laser radar

## Abstract

In the simultaneous localization and mapping (SLAM) process of indoor mobile robots, accurate and stable point cloud data are crucial for localization and environment perception. However, in practical applications indoor mobile robots may encounter glass, smooth floors, edge objects, etc. Point cloud data are often misdetected in such environments, especially at the intersection of flat surfaces and edges of obstacles, which are prone to generating jump points. Smooth planes may also lead to the emergence of misdetected points due to reflective properties or sensor errors. To solve these problems, a two-step filtering method is proposed in this paper. In the first step, a clustering filtering algorithm based on radial distance and tangential span is used for effective filtering against jump points. The algorithm ensures accurate data by analyzing the spatial relationship between each point in the point cloud and the neighboring points, which allows it to identify and filter out the jump points. In the second step, a filtering algorithm based on the grid penetration model is used to further filter out misdetected points on the smooth plane. The model eliminates unrealistic point cloud data and improves the overall quality of the point cloud by simulating the characteristics of the beam penetrating the object. Experimental results in indoor environments show that this two-step filtering method significantly reduces jump points and misdetected points in the point cloud, leading to improved navigational accuracy and stability of indoor mobile robots.

## 1. Introduction

With the rapid development of robotics technology, the application areas of intelligent mobile robots have gradually expanded from traditional fields such as industry and military to the civilian and service sectors. Among these emerging applications, indoor sweeping robots have become one of the most widely used types of indoor mobile robots [1].

Indoor sweeping robots can autonomously perform simultaneous localization and mapping (SLAM), environment traversal, floor sweeping, garbage collection, charging, and other functions in a home environment. They provide a good user experience, becoming a successful example of mobile robots moving from research sites to everyday applications [2]. However, as the application range of sweeping robots continues to expand, their working environments have become more and more complex. Thus, the requirements for their performance have increased, bringing new challenges to technology development. Today, point cloud filtering technology is of the important technical challenges facing indoor sweeping robots.

Point cloud data for sweeping robots consist of a set of data points discretely collected by a laser-ranging sensor on a scanning plane to characterize the distribution of obstacles in the working environment. Due to the influence of ambient light, sensor thermal noise, geometric properties of the ranging environment, optical properties of the obstacle surface, and robot movement, there are inevitably noise and anomalies in the point cloud data; thus, the point cloud data need to be filtered before being used in data analysis and processing [3].

This paper proposes a two-step filtering approach for indoor LiDAR (Light Detection and Ranging) point clouds, targeting the efficient removal of jump points and misdetected points that often disrupt accurate mapping and navigation in mobile robots. The proposed approach utilizes a clustering filtering algorithm based on radial distance and tangential span to remove jump points and a grid penetration model to eliminate misdetected points on smooth surfaces. Compared with the existing methods, our new method improves the accuracy of point cloud filtering for indoor sweeping robots while reducing false deletions and omissions. The main contributions of this paper are as follows:

1. Introduction of a clustering filtering algorithm based on radial distance and tangential span, achieving efficient jump point detection and removal.
2. Implementation of a grid penetration model to filter misdetected points on smooth surfaces, significantly improving data quality and navigation stability.
3. Strong performance and adaptability in complex indoor environments, outperforming existing methods in preserving point cloud details while minimizing noise.

The rest of this paper is structured as follows: Section 2 reviews and categorizes point cloud filtering algorithms for indoor sweeping robots, outlining each approach’s characteristics, strengths, and limitations; Section 3 describes the point cloud data acquisition process and examines causes of anomalous data in various environments; Section 4 introduces the two filtering algorithms—a clustering algorithm based on radial distance and tangential span, and a grid penetration model filter—detailing their implementation and improvements over existing methods; Section 5 presents experiments and results comparing the proposed algorithms with state-of-the-art methods finally, Section 6 concludes the paper by discussing the advantages of the proposed two-step filtering approach.

## 2. Related Work

Point cloud filtering algorithms for sweeping robots can be classified into image-based methods [4], feature-fitting methods [5], local statistical methods [6], and density clustering methods [7].

### 2.1. Image-Based Filtering Algorithms

Image-based filtering algorithms are used to convert point cloud data into a grid map, followed by applying an image processing algorithm to filter the grid map. Ref. [8] converted laser point clouds into grayscale images and used a one-dimensional median filter to smooth out pixels with abnormal grayscale values, which effectively solves the problem of local data loss in the LiDAR point cloud. Ref. [9] used the canny edge detection operator and contour detection operator for point cloud filtering to improve the filtering efficiency of large-scale photon point clouds, but incurred a certain degree of data loss caused by rasterization. These two algorithms encounter difficult in fine denoising of photon point clouds. Ref. [10] adopted the bilateral filtering algorithm, which can perform weighted averaging and iterative steps on sampling points in adjacent regions, thereby achieving pixel smoothing. However, this algorithm is sensitive to the distribution characteristics of point clouds and is not suitable for situations with high noise. Ref. [11] effectively solved the problem of low filtering accuracy in morphological methods when point clouds are discontinuous by introducing Kriging interpolation to calculate curvature changes in different regions. Ref. [12] proposed a hybrid regression model that combines Gaussian process regression and robust locally weighted regression, then divides the filtering process into two stages: radial filtering and circular filtering. The algorithm effectively reduces the influence of outliers on the filtering effect, but can easily lead to edge blurring.

### 2.2. Feature Fitting-Based Filtering Algorithms

Feature-fitting-based filtering algorithms fit the point cloud to geometric features and then filter out outliers and anomalies by observing the differences between the data points and the feature curves. Ref. [13] applied fitting to a number of data points before and after the target point to a straight line, then judged whether the point was a noise point according to the distance from the target point to the straight line. However, the filtering effect of this algorithm is not stable enough when the point cloud presents a nonlinear distribution. Ref. [14] used the least squares method to fit the frame of laser point cloud into a curve and eliminated the noise by judging the distance from the data points to the curve, but its curve fitting process is easily affected by individual outliers and the algorithm can easily fail when there are outliers with a long distance in the point cloud.

### 2.3. Local Statistics-Based Filtering Algorithms

Local statistics-based filtering algorithms calculate the local statistics of each data point as evaluation parameters and then classify the data points into valid points and noise points based on the ranking of these evaluation parameters. Ref. [15] proposed an adaptive method that combines local density and local projection, which can effectively remove sparse outliers, isolated outliers, and non-isolated outliers. For non-isolated outliers, the algorithm performs projection processing by locally fitting a plane, rather than directly removing them. However, this method may result in misjudgment when processing point cloud data with significant density changes, especially in edge or complex surface areas. Ref. [3] proposed an adaptive kernel method that combines local density and global statistical information for point cloud denoising. This method performs statistical analysis on the distance of local points and uses a multivariate Gaussian distribution model to denoise the point cloud while preserving its local features. However, due to its reliance on global statistics and complex kernel density estimation, this method has a heavy computational burden and low efficiency when processing large-scale point cloud data.

### 2.4. Density Clustering-Based Filtering Algorithms

Density clustering-based filtering algorithms calculate the local density of each point, classifying data points into core points, edge points, and outliers. Starting from core points, the point cloud is clustered into several groups; outliers and invalid points are then filtered out by analyzing the clusters. Ref. [16] achieved a better filtering effect by comprehensively taking into consideration the geometrical information of the point cloud when clustering the data points. However, this algorithm can fail when the laser point cloud has missing data. Ref. [17] improved the denoising accuracy of the density-based clustering method by constructing an elevation frequency histogram using empirical mode decomposition, then applying progressive densification. Ref. [18] proposed a clustering method based on OPTICS (Ordering Points To Identify the Clustering Structure) to sort the unordered point cloud and change the density search region to an ellipse. The method is insensitive to the clustering parameters and has good robustness. Ref. [19] proposed a filtering algorithm based on quad-tree isolation, which requires no input parameters and improves the timeliness of the filtering algorithm in large-scale point cloud scenarios.

### 2.5. Deep Learning-Based Filtering Algorithms

Recent years have seen widespread application of deep learning-based methods to research on point cloud filtering. Ref. [4] proposed a LiDAR point cloud filtering method based on a multi-scale convolutional neural network (CNN) combined with an attention mechanism. This transforms the point cloud problem into an image classification task, improving classification accuracy. The method demonstrated superior performance on standard ISPRS datasets and the Qinghai point cloud dataset. Ref. [20] proposed a deep learning framework of geometric perception, which was used to accurately detect the cutting points of plants in the unstructured field environment. By learning the geometric prior knowledge of the point cloud, the accuracy of the task can be significantly improved. Although deep learning methods have shown great potential in high-level semantic understanding and complex tasks, they usually require massive amounts of annotation data for training, leading to huge computational overhead and poor interpretability of the model.

### 2.6. Vision–LiDAR Fusion-Based Filtering Algorithms

Recent advances have explored the integration of visual data with LiDAR point clouds to improve filtering accuracy and robustness. Vision–LiDAR fusion approaches leverage the complementary strengths of cameras (rich texture and color information) and LiDAR (precise depth measurements) to enhance point cloud processing. These methods typically involve projecting 3D point cloud data onto 2D image planes or fusing feature representations from both modalities. A recent study by Ref. [21] presented a novel fusion framework that effectively addresses misdetected points through cross-modal validation, demonstrating significant improvements in complex indoor environments similar to those addressed in our work. While these fusion approaches generally require additional computational resources and careful calibration between sensors, they offer promising directions for future research in point cloud filtering for indoor mobile robots.

In general, the advantage of image-based filtering algorithms lies in the rich variety of algorithms that can be borrowed and the relative maturity of related research. However, the gray value of the image converted from the laser point cloud is usually more monotonous, which is not conducive to giving full play to the advantages of image-based filtering algorithms [22]. Feature-fitting-based filtering algorithms can retain the original data points, and can better maintain the shape and contour; however, this type of algorithm has higher requirements for the quality of the point cloud as well as the sampling density, and the operation speed is slower on large-scale point cloud data [23]. Local statistics-based filtering algorithms do not require high sampling density of the point cloud, but the filtering effect is easily affected by the parameter threshold, resulting in low adaptive performance, and the performance is not robust enough [24]. Density clustering-based filtering algorithms are less dependent on the distribution of the point cloud and system thresholds, but have better adaptability; however, the algorithms in this category need to set up a suitable density search area [25]. Deep learning-based filtering methods utilize a multi-scale convolutional neural network (CNN) combined with an attention mechanism to improve classification accuracy. These methods generally require high computational costs and large datasets for training. Vision–LiDAR fusion-based filtering method can combine the rich texture information of a camera and the precise depth measurement advantages of LiDAR; however, this method requires additional computing resources, increases the complexity of the system, and encounters performance limitations in dynamic environments with few features. In contrast to existing methods, our two-step filtering approach can overcome the limitations of fixed thresholding in local statistics-based methods and preserve the spatial details lost in image-based approaches while avoiding the computational complexity of vision–LiDAR fusion systems. As such, the proposed approach delivers superior performance in filtering both jump points and misdetected points with minimal computational overhead, making it ideal for resource-constrained indoor sweeping robots.

## 3. Point Cloud Data Analysis of Indoor Sweeping Robots

### 3.1. Operating Principle of Laser Range Sensors

**Figure 1:**
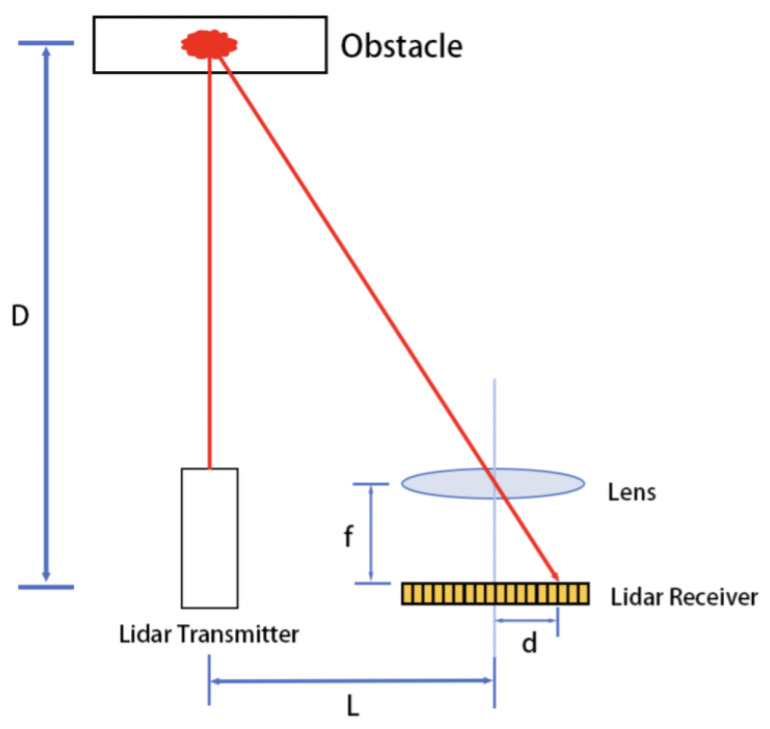
Working principle of a laser range sensor.

Indoor floor sweeping robots detect obstacle information in the environment through ranging sensors. LiDAR dominates the ranging sensors used in indoor floor sweeping robots due to its wide measuring range, simple equipment, low production cost, and other advantages. Figure 1 shows the operating principle of the laser range sensor.

As shown in Figure 1, the transmitter emits a spot laser light, which is reflected by an obstacle and then focused into an image on the receiver through a lens. Here, *L* is the system width, i.e., the perpendicular distance from the center point of the lens to the emitted light ray, *f* is the distance from the lens to the light-sensitive surface, *D* is the distance from the obstacle to the laser ranging sensor, and *d* is the distance of the reflected light spot on the light-sensitive surface.

The measured distance is shown in Equation (1):

(1) $$D=\frac{f\times (L+d)}{d}$$

(2) $$d=l_{pix}\times k$$

where $l_{pix}$ is the length of a single-pixel point on the light-sensitive surface and *k* is the number of pixels corresponding to distance *d* on the light-sensitive surface.

From Figure 1, Equations (1) and (2), it can be seen that when the measured distance *D* is larger, a smaller distance *d* of the spot on the light-sensitive surface results in a wider range of distances corresponding to a single pixel on the light-sensitive surface; in other words, a larger measured distance corresponds to lower sensor ranging accuracy. This feature of the laser ranging sensor makes the point cloud of the sweeping robot present different distribution densities at different distances; thus, problems with mistaken or omitted deletions can occur when using the traditional filtering algorithm.

### 3.2. Point Cloud Data Acquisition

**Figure 2:**
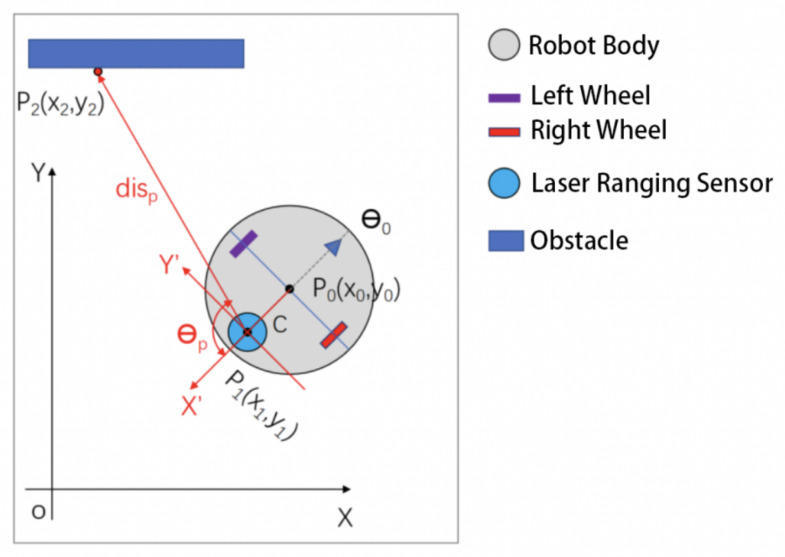
World coordinate system and sensor coordinate system of mobile robot.

The data output from the laser ranging sensor contains the measured distance in the sensor coordinates and the angle of the outgoing light when the distance was obtained; its data format is shown in Equation (3):

(3) $$A=\{\left(\theta_{0},dis_{0}\right),\ldots ,\left(\theta_{i},dis_{i}\right),\ldots ,\left(\theta_{N},dis_{N}\right)\}$$

where $\theta_{i}$ is the outgoing light angle corresponding to the i-th data, $dis_{i}$ is the measured distance, and *N* is the amount of measurement data. The sensor coordinate system relative to the world coordinate system is shown in Figure 2.

In Figure 2, XOY is the world coordinate system, $P_{0}\left(x_{0},y_{0},\theta_{0}\right)$ is the robot’s position, $P_{1}$($x_{1}$,$y_{1}$) is the sensor center, *C* is the distance from the robot center to the sensor center, $X^{\prime }OY^{\prime }$ are the ranging sensor coordinates, $P_{2}\left(x_{2},y_{2}\right)$ is the sampling point, $\theta_{p}$ is the corresponding outgoing light angle, and $dis_{p}$ is the corresponding measured distance.

The coordinates of the sampling point can be calculated from Equation (4):

(4) $$\left\{\begin{array}{c} x_{1}=x_{0}-C\times cos\theta_{0}\\ y_{1}=y_{0}-C\times sin\theta_{0}\\ x_{2}=x_{1}+dis_{p}\times cos\left(\theta_{0}+\theta_{p}+\theta_{c}\right)\\ y_{2}=y_{1}-dis_{p}\times sin\left(\theta_{0}+\theta_{p}+\theta_{c}\right) \end{array}\right.$$

where $\theta_{c}$ is the angle of the zero-degree direction of the sensor coordinate system in the world coordinate system. For the indoor sweeping robot used in this paper, $\theta_{c}=-180{}^{\circ}$.

### 3.3. Jump Points and Misdetected Points

#### 3.3.1. Jump Points

**Figure 3:**
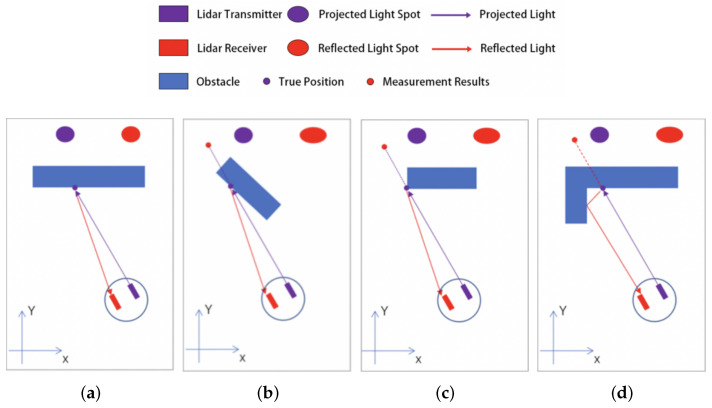
Examples under different incidence angles: (**a**) normal conditions, (**b**) large angle of incidence, (**c**) edges of obstacles, and (**d**) planar joints.

The laser range sensor projects a light spot through the transmitter, which is reflected by the surface of the obstacle and then collected by the receiver. The processor calculates the distance to the obstacle according to the center position of the reflected light spot. In some specific areas in the indoor environment, the projected light spot will be deformed and missing after irradiating the surface of the obstacle, which will have an impact on the measurement results, as shown in Figure 3.

As shown in Figure 3a, under normal circumstances, the projected light spot shines on the surface of the obstacle to produce a reflected light spot. The shape of the reflected light spot is basically the same as that of the projected light spot, and the sensor works normally. As shown in Figure 3b, in the case of a large incident angle, the reflected light spot deforms and the center position of the light spot shifts, resulting in incorrect measurement results. In Figure 3c, the reflected spot shows a similar deformation as in Figure 3b due to the curved surface at the edge of the obstacle, which leads to sensor calculation errors. As shown in Figure 3d, due to the multiple reflections of the light spot in the local range, the shape and position of the light spot at the planar connection are changed, which makes it more difficult to determine the measurement results. All of the above three scenarios are prone to measurement errors, resulting in jump points in the point cloud.

In this work, we define a *jump point* as a LiDAR return that shows an abnormal range discontinuity with its adjacent beam such that the Euclidean distance between consecutive points exceeds a continuity threshold or the cluster formed around it covers an angular span smaller than the minimum expected span at that distance. The explicit quantitative criteria are provided in Equations (5)–(7).

#### 3.3.2. Misdetected Points

**Figure 4:**
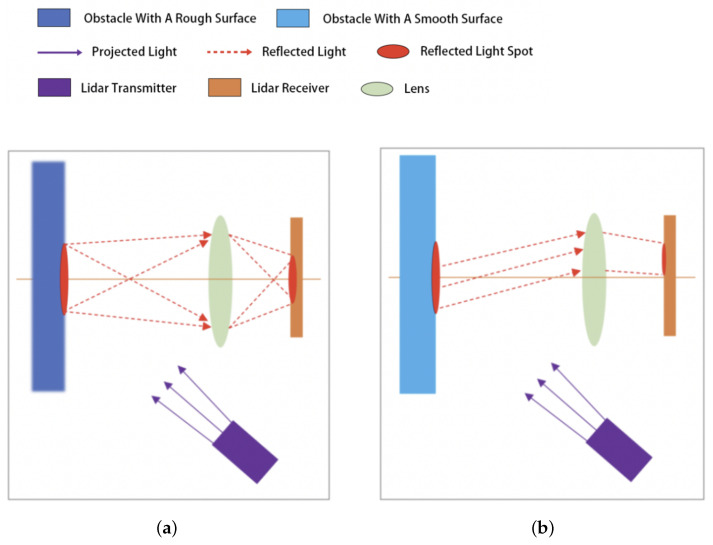
Reflected light on different surfaces: (**a**) obstacles with rough surfaces and (**b**) obstacles with smooth surfaces.

In addition to the geometric shape, the reflection characteristics of the obstacle surfaces may also have an impact on the measurement results. Indoor environments generally contain some smooth surfaces on which the scattering characteristics of the light spot are altered; as shown in Figure 4, this can affect the calculation results of the sensor. This phenomenon exemplifies a fundamental challenge that permeates various 3D sensing applications, namely, accurate surface detection and representation. For instance, ref. [26] presented a method for super-resolved detection of surfaces in volumetric fluorescence microscopy, demonstrating how surface characteristics fundamentally influence measurement outcomes across different sensing modalities. Although this method operates in the domain of fluorescence microscopy, while our work focuses on indoor robot navigation with LiDAR, both face the core challenge of accurately detecting and representing 3D surfaces despite their differing physical principles. This further validates the view that the challenges encountered in handling misdetected points caused by smooth surface reflection properties are not isolated to our specific application, but represent a broader issue in 3D sensing systems.

As shown in Figure 4a, when the light spot passes through the rough surface of the obstacle and undergoes diffuse reflection, it passes through the lens to form a projection on the light-sensitive surface. The sensor is able to accurately calculate the distance to the obstacle by analyzing the center position of the projection.

In Figure 4b, when projected on a smooth surface, the spot lacks sufficient diffuse reflection and the light is mainly concentrated in a certain direction by specular reflection. This shifts the spot’s projected position on the light-sensitive surface, leading to sensor calculation errors and misdetected points in the point cloud.

## 4. Point Cloud Filtering Algorithms for Indoor Sweeping Robots

To address at the problem of indoor sweeping robot point cloud data being prone to jump points at plane intersections and obstacle edges as well as misdetected points at smooth planes, this paper proposes a two-step filtering method. First, a clustering filtering algorithm based on radial distance and tangential span is used to remove the jump points; second, a filtering algorithm based on the grid penetration model is used to further filter out the misdetected points.

### 4.1. Clustering Filtering Algorithm Based on Radial Distance and Tangential Span

From the analysis in Section 2, it can be seen that the laser ranging sensor is prone to wrong measurement results in special cases such as large incidence angles, obstacle edges, and plane junctions, which leads to jump points in the point cloud. For this reason, we propose a clustering filtering algorithm based on the radial distance and tangential span; the radial distance is the distance from the data point to the sensor, while the tangential span is the crossing angle of the projected light corresponding to the data point.

A frame of point cloud data is denoted as *P* = $\left\{p_{1},p_{2},\ldots ,p_{N}\right\}$, where *N* is the number of ranging points, $p_{i}\left(x_{i},y_{i},\theta_{i},d_{i}\right)$ denotes the i-th data point, $\left(x_{i},y_{i}\right)$ is the coordinate of the data point, $\theta_{i}$ is the angle of outgoing light corresponding to the data, and $d_{i}$ is the corresponding measured distance.

The steps of the algorithm are as follows:

1. In the initial state, all data points are free points.
2. Select the free point with the smallest serial number $p_{i}\left(x_{i},y_{i},\theta_{i},d_{i}\right)$ and mark it as a non-free point.
3. Set the proximity point distance threshold $dth_{i}$. From the analysis in Section 3.1, it can be seen that the point cloud data have different densities at different measured distances; the larger the measured distance, the lower the data density. A fixed distance threshold will cause the data points nearest to the robot to be over-adopted and those farthest away to be over-filtered. For this reason, in this paper the proximity distance threshold is dynamically set according to the radial distance of the data points, as shown in Equation (5):
  (5) $$dth_{i}=\frac{2\times d_{i}\times sin\left(\Delta \theta \right)\times \alpha }{cos(\theta_{K})}$$
  where $d_{i}$ is the measured distance corresponding to the data point, Δθ is the angular accuracy of the sensor, $\theta_{K}$ is the maximum angle allowed between the surface of the obstacle and the tangent direction of the projected light, and α is the magnification factor, as shown in Figure 5a.

**Figure 5:**
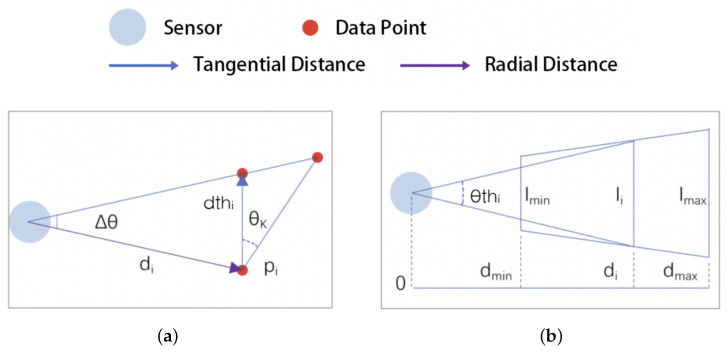
Reflected light on different surfaces: (**a**) proximity distance thresholds and (**b**) span thresholds.
4. Search for free points within a distance less than $dth_{i}$ from $p_{i}$, group them into a point cluster with $p_{i}$, and mark them as non-free points.
5. Repeat steps 2 to 4 for all points in the point cluster to obtain the point cluster *C* = $\left\{q_{1},q_{2},\ldots ,q_{M}\right\}$, where *M* is the number of points and $q_{i}\left(x_{i},y_{i},\theta_{i},d_{i}\right)$ denotes the i-th data point in the point cluster.
6. Calculate the tangential span $\theta_{C}$ of the point clusters. Traditional filtering algorithms use the number, distance, or density of data points as indicators to judge the point clusters; however, in indoor sweeping robot application scenarios these indicators are very likely to be invalidated due to the influence of factors such as the distance and alignment direction of sampling points. For this reason, in this paper we calculate the tangential span of point clusters as an evaluation index. This is shown in Equation (6), where $\theta_{i}$ and $\theta_{j}$ are the respective angles of outgoing light corresponding to data points *i* and *j*:
  (6) $$\theta_{C}=\max_{0<i<M}\left(\max_{1<j\leq M}\left(\theta_{i}-\theta_{j}\right)\right).$$
7. Set the span threshold $\theta th_{i}$ as shown in Equation (7):
  (7) $$\theta th_{i}=2\times arctan\left(\frac{l_{min}+\left(l_{max}-l_{min}\right)\times \left(d_{i}-d_{min}\right)/\left(d_{max}-d_{min}\right)}{2\times d_{ave}}\right)$$
  where $d_{max}$ is the maximum measurement distance of the sensor, $d_{min}$ is the minimum measurement distance of the sensor, $l_{max}$ is the minimum tangential span allowed at the maximum measured distance position, $l_{min}$ is the minimum tangential span allowed at the minimum measured distance location, and $d_{ave}$ is the average measured distance of all data points in the point cluster, as shown in Figure 5b.
8. If $\theta_{C}\geq \theta th_{i}$, then the data points in the point cluster are determined to be valid; otherwise, they are deleted.
9. Repeat steps 2 to 8 until there are no more free points in the point cloud.

### 4.2. Point Cloud Filtering Algorithm Based on Grid Penetration Modeling

From the analysis in Section 3.3, it can be seen that the diffuse reflection characteristics of the projected spot on a smooth obstacle surface are changed, which can easily lead to misdetected points in the point cloud. For this reason, in this paper we propose a point cloud filtering algorithm based on the grid penetration model. The basic idea is to filter the latest frame of the point cloud with the help of the statistics of the recent frames of the point cloud.

The steps of the algorithm are as follows:

1. Initialization (frame count $F_{tot}=0$). Create a cache array for caching the point cloud of each frame over a period of time.
2. Acquire a new frame of point cloud; add 1 to the frame count $F_{tot}$.
3. Grid update. Generate a grid map with the grid width set as $W_{g}$ (in the experiment reported in this paper, $W_{g}=$ 5 cm). Map the data points in the cache array to the grid space sequentially, and add 1 to the corresponding grid count for each mapping.
4. Point cloud caching. Add a new frame point cloud to the point cloud cache and record the time corresponding to the frame point cloud. For data frames in the cache pool, if the survival time is greater than $T_{max}$, removed it from the cache to maintain the timeliness of the cached data points, with $T_{max}$ as the set maximum survival time.
5. Frame count judgment. The initial period of the robot’s work is $F_{tot}<FL_{min}$. If there is not enough statistical information at this time, jump to step 2. If $F_{tot}\geq FL_{min}$, this means that the statistical information is sufficient; then, continue to the next step. Here, $FL_{min}$ is the minimum number of frames required to execute the next step.
6. Penetration modeling. Map grids with a count value greater than $obs_{th}$ are considered obstacle grids, and are denoted as $Q=\{q_{1},q_{2},...,q_{N}\}$. Here, $obs_{th}$ is the set threshold of obstacle counts, $q_{n}$ is the position of the grid center in the world coordinate system, and *N* is the number of obstacle grids.
  Take a data point from the latest frame of the point cloud $p_{i}$, calculating its relationship to the obstacle grid $q_{n}$ and the robot $r_{k}$’s relative position relation, as shown in Figure 6.

**Figure 6:**
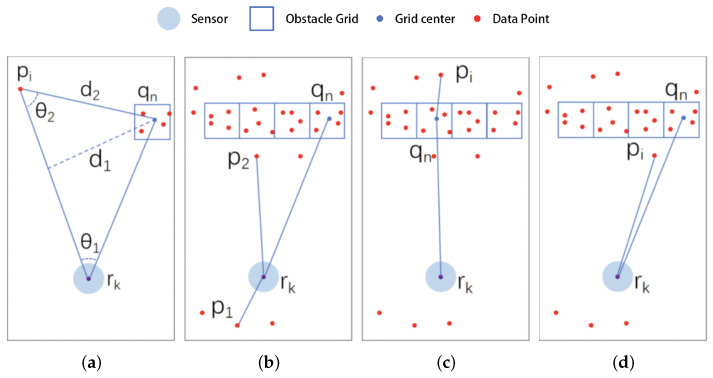
Penetration model: (**a**) parameters, (**b**) no obstacles in the way, (**c**) penetration of obstacles, (**d**) no obstacles in the way.
  In Figure 6a, the angle between $\vec{pr}$ and $\vec{rq}$ is denoted as $\theta_{1}$, the angle between $\vec{pr}$ and $\vec{pq}$ is denoted as $\theta_{2}$, the distance from obstacle $q_{n}$ to $\vec{pr}$ is denoted as $d_{1}$, and the distance between data point $p_{i}$ and obstacle $q_{n}$ is denoted as $d_{2}$.
  For data point $p_{i}$, we perform the following judgment operations:
  (a) Remove the obstacle grid points $q_{n}$.
  (b) Calculate $\theta_{1}$. If $|\theta_{1}|\;>90$, i.e., if the data point is not on the same side of the robot as the obstacle (as shown by $p_{1}$ in Figure 6b), then skip to step 6.
  (c) Calculate $d_{1}$. If $d_{1}>\sqrt{2}\times W_{g}$, i.e., if the obstacle does not shade the transmitted light (as shown by $p_{2}$ in Figure 6b), then skip to step 6.
  (d) Calculate $\theta_{2}$. If $|\theta_{2}|\;\leq 90$, i.e., if the projected light traverses the obstacle grid and $p_{i}$ is judged to be a misdetected point (as shown in Figure 6c), then skip to step 7.
  (e) Calculate $d_{2}$. If $d_{2}<\sqrt{2}\times W_{g}$, i.e., if the data point is too close to the obstacle grid, then $p_{i}$ is determined to be a misdetected point, as shown in Figure 6d. In this case, skip to step 7.
  (f) Repeat steps (a) to (e) until all obstacle grids are compared.
7. Repeat step 6 until all data points in the new frame have been processed.
8. Repeat steps 2 to 6 until all data frames are processed.

### 4.3. Comparison with Existing Filtering Methods

In this work, we have selected two representative baseline filtering algorithms that are commonly used in indoor LiDAR processing. This choice ensures a fair comparison that directly reflects the effectiveness of the proposed method on the two targeted noise types (jump points and misdetected points). Although more general state-of-the-art methods such as statistical outlier removal and learning-based denoising exist, they are not specifically optimized for these indoor noise sources. Extending the comparison to such methods will be an important direction for future work.

#### 4.3.1. Comparison of the Proposed Clustering Filtering Algorithm with the State-of-the-Art

Zhu et al. [18] proposed a clustering filtering algorithm called OPTICS (Ordering Points To Identify the Clustering Structure) to sort the unordered point cloud and change the density search region to an ellipse. This algorithm is the most recent clustering-based point cloud filtering algorithm known to the authors.

**Figure 7:**
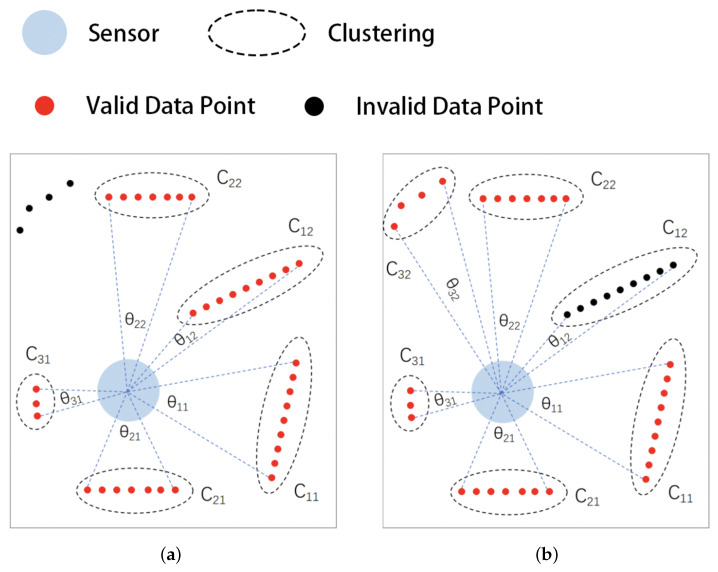
Comparison between OPTICS clustering-based filtering algorithm and the proposed algorithm: (**a**) OPTICS and (**b**) proposed filtering algorithm.

Figure 7 shows the comparison between the OPTICS algorithm and our proposed clustering filtering algorithm. In the figure, the data points within the elliptical dashed line form the point clusters, with red dots indicating valid data points and black dots indicating invalid data points; $C_{11}$ To $C_{32}$ are the numbers of the point clusters, and $\theta_{11}$ to $\theta_{32}$ is the span of the point cluster.

As shown in Figure 7a, the OPTICS algorithm judges the point clusters based on the density of the data points in the local area. The four data points in the upper left corner are judged as invalid points due to their spacing, which means that they cannot form point clusters with other data points and that the density of the point cloud is low. The remaining five point clusters have localized densities that meet the requirements, and as such are judged as valid points.

As shown in Figure 7b, the filtering algorithm proposed in this paper dynamically adjusts the clustering threshold according to the distance from the data points to the sensor (Equation (5)). The four points in the upper left-hand corner form a point cluster $C_{32}$ and their spans meet the requirements; thus, they are judged as valid points. For point cluster $C_{12}$, although its data points are sufficiently large and dense enough, its span relative to the sensor is small; therefore, it is judged to be an invalid point.

From the above analysis, it can be seen that the proposed clustering filtering algorithm based on radial distance and tangential span dynamically adjusts the proximity point clustering threshold and the point cluster span threshold according to the radial distance of the data points, then adopts the point cluster span instead of the local density to judge the point clusters. In the application scenario of indoor sweeping robots, the proposed filtering algorithm can more effectively avoid the problems of mistaken deletion and omission when compared to the existing OPTICS algorithm.

#### 4.3.2. Comparison of the Proposed Grid Penetration Model-Based Filtering Algorithm with the State-of-the-Art

Wang [8] proposed an image-based point cloud filtering algorithm that converts a laser point cloud into a grayscale image and uses a median filter to smooth pixels with abnormal grayscale values. This algorithm is the latest image-based point cloud filtering algorithm known to the authors.

**Figure 8:**
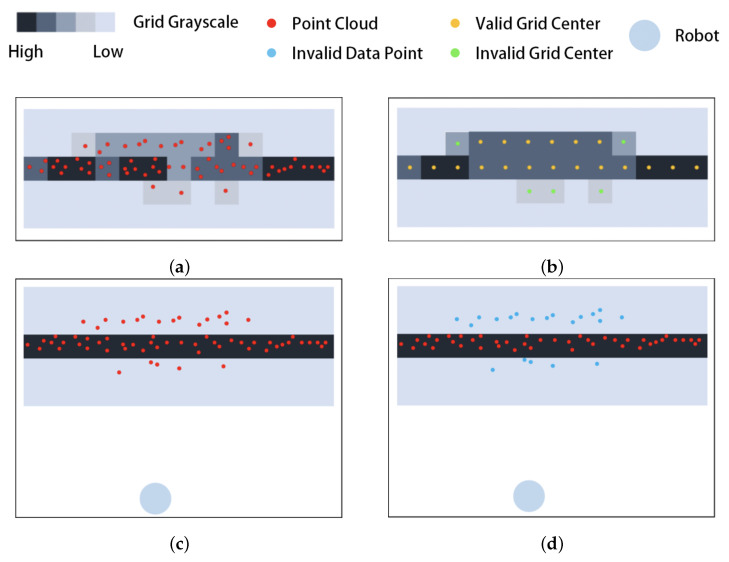
Comparison of existing image-based filtering algorithm with the proposed grid penetration model-based algorithm: (**a**) raw point cloud and grid grayscale, (**b**) filtering results of existing algorithm, (**c**) raw point clouds and obstacle grids, (**d**) filtering results of our proposed algorithm.

Figure 8 shows the comparison between the existing algorithm and our proposed filtering algorithm based on the grid penetration model.

Figure 8a shows the original point cloud and the grayscale image obtained from the conversion; as shown in Figure 8b, the existing algorithm uses a median filtering method to adjust the grid grayscale, where the grayscale above the threshold is considered valid and its midpoint is used as the data point (shown by the yellow dot in the figure), while the grayscale below the threshold is considered invalid (shown by the green dot in the figure).

As shown in Figure 8c, our filtering algorithm based on the grid penetration model obtains the obstacle grid based on the original point cloud statistics (shown by the black grid in Figure 8d). Figure 8d shows that the proposed algorithm filters the original data based on the transmission model, retaining the data points that satisfy the penetration relationship (shown by the red dots in Figure 8c) and deleting those that do not (shown by the blue dots in Figure 8d).

From the above analysis, it can be seen that the existing algorithm adopts pixel median filtering, which can filter out part of the noise and make the edges smoother, but cannot filter out misdetected points caused by smooth planes. The existing method replaces the original data points with the center of the grid, resulting in part of the details being lost. In contrast, our proposed filtering algorithm based on the grid penetration model takes into account the relative positions of the data points, the robot, and the obstacle grids, allowing it to effectively filter out misdetected points while retain the details of the data. This enables the indoor sweeping robot to carry out the subsequent tasks of straight line extraction, map construction, and path planning.

## 5. Experimental

### 5.1. Experimental Environment

**Figure 9:**
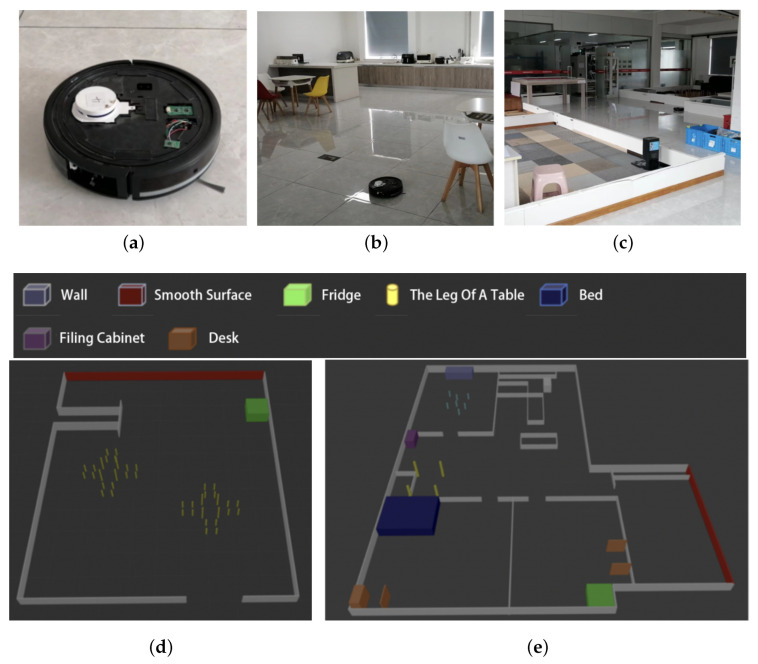
The indoor sweeping robot and testing environment: (**a**) indoor sweeping robot, (**b**) experimental environment I, (**c**) experimental environment II, (**d**) environment I simulation model, and (**e**) environment II simulation model.

The experiment used a sweeping robot platform to collect point cloud data and implemented the online version of the proposed filtering algorithm is on the same platform. To analyze and demonstrate the algorithm process more intuitively, the offline version of the algorithm was implemented on the ROS system. The sweeping robot platform and experimental environment are shown in Figure 9.

As shown in Figure 9a, the indoor sweeping robot has a circular shape, with a diameter of about 34 cm and a height of about 12 cm. the robot obtains information about obstacles by means of a laser ranging sensor with angular accuracy Δθ=1°, minimum measurement distance $d_{min}=15$ cm, and maximum measurement distance $d_{max}=600$ cm.

As shown in Figure 9b, experimental environment I consists of an area of about 8 m × 10 m = 80 $m^{2}$ of the hall, which is furnished with chairs, refrigerators, and other furniture. A smooth building facade exists for the wall located above the picture.

Figure 9c shows experimental environment II. This environment simulates a home space, with a total area of about 15 m × 18 m = 270 $m^{2}$. It consists of four “rooms” and a “hall” with common furniture such as beds, sofas, tables, chairs, filing cabinets, refrigerators, etc. The wall on the right side of the picture consists of smooth metal strips, while the wall on the right side of the picture consists of smooth metal bars.

Figure 9d and Figure 9e show the simulation models of environment I and environment II, respectively.

### 5.2. Experiments on Clustering Filtering Algorithms Based on Radial Distance and Tangential Span

The clustering filtering algorithm based on radial distance and tangential span was experimented with and compared with the clustering filtering algorithm proposed by Zhu et al. [18] in two different indoor environments.

**Figure 10:**
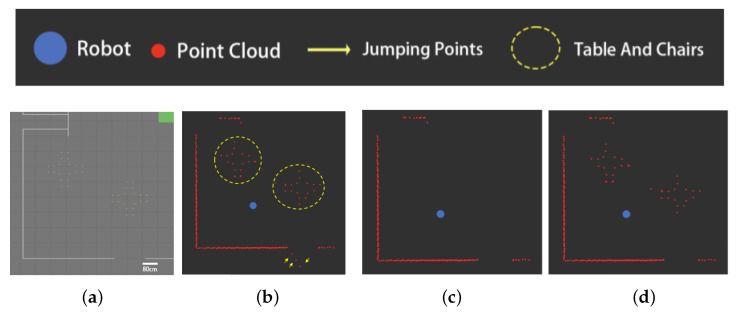
Testing results of the clustering-based filtering algorithm in environment I: (**a**) parameters, (**b**) no obstacles in the way, (**c**) penetration of obstacles, (**d**) no obstacles in the way.

Figure 10 shows the experimental results of the clustering filtering algorithms in environment I.

As can be seen from Figure 10, the sweeping robot point cloud contains jump points at the edge of the obstacle (shown by yellow arrows). While the existing algorithm is able to filter out the jump points (Figure 10c), the algorithm bases its judgment on the density and number of point clouds; thus, the more sparsely distributed table and chair data points (shown by the yellow circles) are mistakenly deleted.

As shown in Figure 10d, the clustering filtering algorithm proposed in this paper dynamically sets the clustering threshold according to the radial distance between the data points and the robot, and is based on the point cluster span. Thus, the table and chair data points are retained, as their point cluster span meets the requirements even though they are more sparsely distributed.

**Figure 11:**
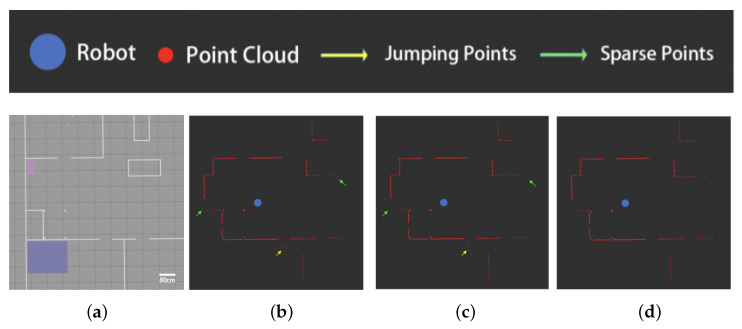
Testing results of the clustering-based filtering algorithms in environment II: (**a**) parameters, (**b**) no obstacles in the way, (**c**) penetration of obstacles, (**d**) no obstacles in the way.

Figure 11 shows the experimental results of the clustering filtering algorithms in environment II.

As shown in Figure 11, the distribution of measured points farther away from the robot is sparse (shown by green arrows), which the existing algorithm considers invalid points to be deleted (Figure 11c). Meanwhile, several consecutive jump points at the edge of the obstacle (shown by yellow arrows) are incorrectly retained due to their localized density.

In contrast, the clustering filtering algorithm proposed in this paper dynamically adjusts the clustering threshold according to the radial distance between the data points and the robot, which avoids the problem of erroneous deletion of long-distance data points. Because the clustering algorithm proposed in this paper is based on the point cluster span, a number of jump points at the edge of the obstacle are judged to be invalid due to their small point cluster span, avoiding the problem of missed deletions.

### 5.3. Experiments on Filtering Algorithm Based on the Grid Penetration Model

The filtering algorithm based on the grid penetration model was experimented with to compared it with the image-based filtering algorithm proposed by Wang [8] in two different indoor environments.

**Figure 12:**
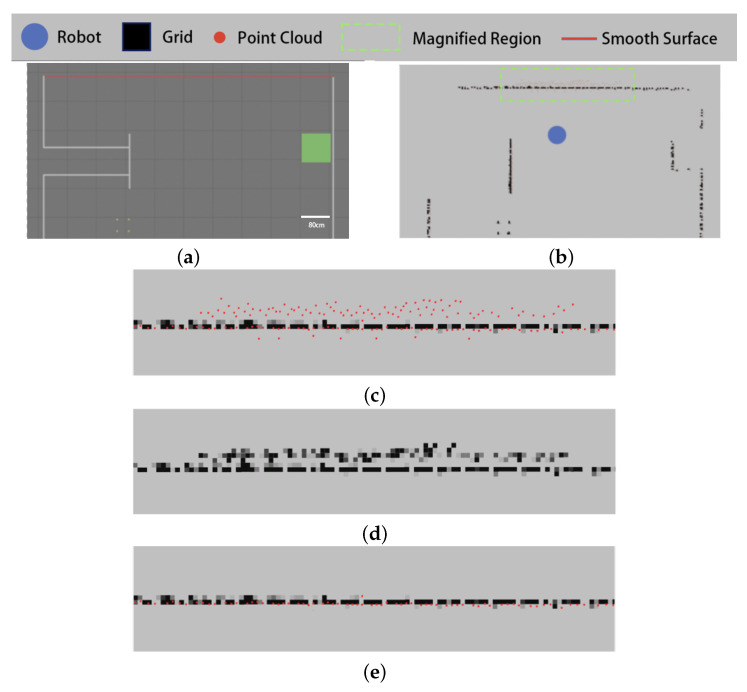
Testing results of the image-based filter in environment I: (**a**) topology of environment I, (**b**) raw point clouds and grid maps, (**c**) localized enlargement of the original point cloud, (**a**) filtering results of the existing algorithm, and (**e**) filtering results of the proposed algorithm.

The results of the experiment in the first indoor environment are shown in Figure 12.

As shown in Figure 12c, the point cloud of the sweeping robot includes misdetected points on smooth surfaces. The existing filtering algorithm counts the point cloud as a grid map, then adopts the median value of the grid grayscale in the local window as the grayscale value of the target grid.

As shown in Figure 12d, while the existing algorithm filters out some of the more sparsely distributed misdetected points, most of the misdetected points cannot be filtered out correctly, and the obstacle contours in the grid map are changed.

In contrast, our proposed filtering algorithm based on the grid penetration model filters the point cloud according to the relative positional relationship between the data points, obstacle grids, and sensors. As shown in Figure 12e, this allows it to correctly filter out the misdetected points generated by smooth surfaces.

**Figure 13:**
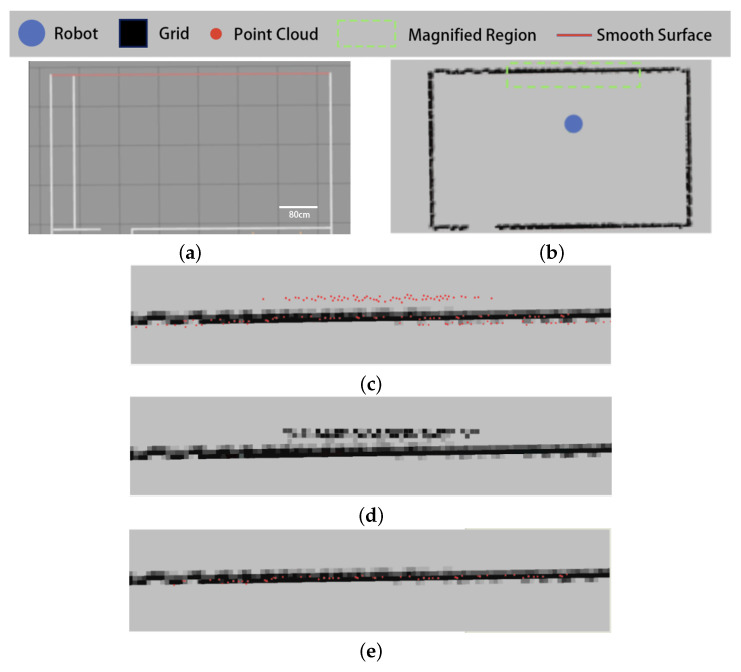
Testing results of the image-based filter in environment II: (**a**) topology of environment II, (**b**) raw point clouds and grid maps, (**c**) localized enlargement of the original point cloud, (**d**) filtering results of the existing algorithm, and (**e**) filtering results of the proposed algorithm.

The results of the experiment in the second indoor environment are shown in Figure 13.

As can be seen from Figure 13, there are smooth surfaces in the experimental environment (shown by the red lines), and the sweeping robot point cloud appears to have misdetected points on the smooth surfaces (Figure 13b,c).

Figure 13d,e show the filtering results of the existing algorithm and the algorithm proposed in this paper, respectively. Similar to the results in environment I, the existing algorithm is unable to filter out the misdetected points that appear on the smooth surface in environment II, while the proposed algorithm obtains the correct filtering results.

It is worth noting that the proposed algorithm naturally adapts to different LiDAR resolutions through the dynamic threshold mechanism. As shown in Equation (5), the distance threshold $dth_{i}$ for neighboring points is directly related to the sensor’s angular accuracy Δθ, which allows for automatic adjustment when the resolution changes. Regarding noise levels, the proposed two-step filtering strategy is specifically designed for typical indoor artifacts: (1) a clustering method based on radial distance and tangential span removes jump points at obstacle edges and planar junctions, and (2) a grid-penetration model eliminates false measurements caused by reflections on smooth surfaces. Therefore, although systematic experiments under varying noise conditions were not conducted, both theoretical analysis and validation in diverse indoor scenarios demonstrate that the proposed method exhibits strong adaptability and robustness across different resolutions and noise characteristics.

## 6. Conclusions

This paper proposes a two-step filtering algorithm to address the issues of jump points and misdetected points in indoor LiDAR point cloud data. Compared with existing methods, the core innovation of this work lies in two aspects. First, we propose a clustering-based filtering algorithm based on radial distance and tangential span, which overcomes the limitations of traditional density-based clustering methods. By introducing a dynamic threshold mechanism (Equation (5)) and tangential span evaluation (Equations (6) and (7)) instead of conventional point cloud density criteria, our proposed algorithm can adapt to point cloud density variations at different measurement distances. As illustrated in Figure 10 and Figure 11, this effectively addresses the issues of false deletion in sparse regions and missed deletion at obstacle edges encountered by existing clustering algorithms such as OPTICS [18]. Second, we introduce an innovative grid penetration-based filtering algorithm. This model simulates the physical penetration characteristics of laser beams to precisely analyze the spatial geometric relationships among data points, obstacle grids, and the robot, thereby effectively identifying and filtering false detections caused by reflections on smooth surfaces. This approach fundamentally differs from image transformation methods such as the one in [8] in that it preserves the spatial details of the original point cloud. As confirmed by the results of our comparative experiments in Figure 12 and Figure 13, this avoids information loss and edge blurring caused by rasterization. Experimental results demonstrate that this two-step filtering strategy not only significantly improves point cloud data quality but also provides indoor mobile robots with more accurate environmental perception, showing superior adaptability and robustness in challenging scenarios involving glass, polished floors, and obstacle edges.

## Data Availability

The datasets generated and analyzed during the current study are not publicly available due to privacy restrictions, but are available from the corresponding author on reasonable request.
